# Expert Opinions on Web-Based Peer Education Interventions for Youth Sexual Health Promotion: Qualitative Study

**DOI:** 10.2196/18650

**Published:** 2020-11-24

**Authors:** Philippe Martin, Corinne Alberti, Serge Gottot, Aurelie Bourmaud, Elise de La Rochebrochard

**Affiliations:** 1 Institut National de la Santé Et de la Recherche Médicale (INSERM) Epidémiologie clinique et évaluation économique appliquées aux populations vulnérables (ECEVE) Université de Paris Paris France; 2 Institut National d’Etudes Démographiques (INED), UR14 – Sexual and Reproductive Health and Rights Aubervilliers France; 3 Centre de Recherche en épidémiologie et Santé des Populations (CESP) - UMR U1018 (UVSQ/INSERM) Université Paris-Saclay, Université Paris-Sud Villejuif France; 4 GDID Santé Paris France

**Keywords:** youth, health promotion, internet, sexual health, peer education

## Abstract

**Background:**

Participatory education, in the form of peer education, may be an effective way to promote youth sexual health. With the advent of the internet, web-based interventions have potential as an attractive new tool for sexual health promotion by peers.

**Objective:**

The aim of this study was to evaluate professional experts’ opinions on the perspectives for web-based participatory interventions to promote sexual health by peers and among young people.

**Methods:**

Semistructured interviews were carried out with 20 experts (stakeholders in direct contact with young people, researchers, and institutional actors) specializing in sexual health, health promotion, peer education, youth, internet, and social media. After coding with N’Vivo, data were subjected to qualitative thematic analysis.

**Results:**

The majority of experts (18/20, 90%) found this kind of intervention to be attractive, but highlighted the necessary conditions, risks, and limitations attached to developing an acceptable peer intervention on the internet for sexual health promotion among young people. Five main themes were identified: (1) an internet intervention; (2) sexual health; (3) internet skills, and uses and the need for moderation; (4) multifaceted peers; and (5) minority peers. In the absence of youth interest for institutional messages, the experts highlighted the attractive participatory features of web-based interventions and the need for geolocalized resources. However, they also warned of the limitations associated with the possibility of integrating peers into education: peers should not be mere messengers, and should remain peers so as not to be outsiders to the target group. Experts highlighted concrete proposals to design an online participatory peer intervention, including the process of peer implication, online features in the intervention, and key points for conception and evaluation.

**Conclusions:**

The experts agreed that web-based participatory interventions for youth sexual health promotion must be tailored to needs, uses, and preferences. This type of action requires youth involvement framed in an inclusive and holistic sexual health approach. Peer education can be implemented via the internet, but the design of the intervention also requires not being overly institutional in nature. Involving young people in their own education in an interactive, safe online space has the potential to develop their empowerment and to foster long-term positive behaviors, especially in the area of sexual health.

## Introduction

UNESCO (United Nations Educational, Scientific and Cultural Organization) emphasizes that adolescence is the time to develop healthy habits and lifestyles related to sexual health, when individuals are exploring their sexuality and establishing interpersonal relationships [1]. Young people have emerging questions about sexual health, which go beyond disease and integrate emotional dimensions [2,3]. Major sexual and reproductive health issues affect young people, including puberty, sexually transmitted infections [4], unwanted pregnancy [5], and sexual violence, but information/communication technologies also have an influence on sexual behavior [1].

To address these issues, top-down sexual health education has been developed. However, learning is not limited to receiving and processing information: young people learn best when they are allowed to build their own understanding of information [1]. Among young people, interactive models that promote social interaction and exchanges of experiences could be effective in acquiring this knowledge and developing positive health behavior over the long term [6]. In this process, peer education corresponds to an educational approach that uses peers (sharing the same age, social context, function, education, or experience) to provide information and to promote certain types of behaviors and values [7,8]. Through informal social learning, peers appear as resources offering support and sharing similar experiences. Health actors have tried to advance peer education to become a formal process in public policy and practice, particularly with respect to youth sexual health [9-12].

In the past, peer education programs have been implemented in physical life, especially for HIV prevention and sexual health education [9,13,14], with different peer-led group sessions [9]. Past experiences have highlighted the value of a comfortable and user-friendly space to exchange information and perspectives about sexuality, with peer educators facilitating youth engagement [15]. A recent review of college campus peer interventions found positive improvements in knowledge and behaviors such as condom use and HIV testing [16].

With the advent of new technologies, the internet offers wide access to health information, particularly in sexual health [3], with benefits of interactivity and personalization of information [17]. Social media offer users the ability to generate, share, and receive information through multidirectional exchanges, which can transcend geographical boundaries and provide anonymity in discussing intimate topics [18,19]. Young people can join online communities to benefit from social support and find responses to their concerns. The online media have potential to offer great opportunities for peer education interventions in sexual health for young people.

Despite these recognized benefits, there is little research evaluating participatory interventions on the internet and by peers in youth sexual health. One intervention study explored the feasibility of peer education among adults focusing on men who have sex with men (MSM) for HIV prevention, with the training of leaders in a Facebook group [20]. Another study designed a social media “peer-led” intervention in a Facebook group for safer sex practices among young people [21]. Although the feasibility of these interventions has been demonstrated, much work remains to be done to determine whether this educational model is applicable and effective, and if it is complementary to traditional top-down systems. In particular, further research is needed to explore the educational and practical potential of these interventions, examining inherent risks and limitations. Feedback from experts (in the fields of internet, youth, and sexual education) should make it possible to address several of the key methodological issues.

Community-based participatory research brings together partners (actors in the field, designers and researchers, target audience) with different skills, knowledge, and expertise to address complex problems, including experienced professionals [22]. Collecting their views based on their experiences should inform the design of in-depth analyses, particularly for the development of peer education interventions on youth sexual health [15]. Currently, there are no data available on relevant stakeholders’ opinions and experiences on peer education, sexual health, youth, and the internet. Therefore, there is a need for informative research to fill these gaps, and to develop this new kind of intervention effectively.

Accordingly, the aim of this study was to evaluate expert opinions and to collect advice on web-based participatory interventions to promote young people’s sexual health through peers, and to study the inherent risks and limitations.

## Methods

### Design

Our methodology followed the Consolidated criteria for reporting qualitative research (COREQ) checklist for writing and reading qualitative research reports [23] (see Multimedia Appendix 1).

### Participant Recruitment

We identified 37 French experts on the subject from existing publications in which they were named as authors, as well as reports and health promotion programs in which they were credited as editors and managers. These experts had different functions in diverse fields of expertise, including sexual health, young people’s health, connected health, peer education, and program methodology. They all had concrete experience on the subject and had expertise related to some or all of the study topic. Some of the experts were in charge of websites promoting the sexual health of young people, were developing educational programs or national sexual health guidelines, or had studied the sexual health of young people, particularly through the internet. We invited these experts to participate in the study by email. Of the 37 identified experts, 20 agreed to participate. The characteristics of the participant experts are presented in Table 1 (see Multimedia Appendix 2 for more details).

**Table 1:** Expert characteristics (N=20).

Characteristic		Participants, N	
**Gender**			
	Female		13
	Male		7
**Organization type**			
	Association		5
	Public institute for prevention/health promotion		5
	Public hospital		4
	National education		2
	Public research institute		2
	Public scientific/cultural/professional institution		1
	Government agency		1
**Occupation**			
	Professor of health (public health, health promotion, gynecology)		3
	Nurses and midwives specializing in sexual health		3
	Clinical psychologists/youth psychotherapists		2
	Heads of prevention department/prevention project manager		2
	Sociologist experts on online youth sexuality		2
	College teacher (science and sexuality education)		1
	Regional health education program coordinator		1
	Prevention facilitator; specialized educator		1
	Advisor in social and family economy		1
	Social marketing expertise manager		1
	Documentation and information officer		1
	Epidemiologist		1
	Social worker		1
**Specializations**			
	Youth health		18
	Sexual health		16
	Education/prevention/promotion		12
	Peer education		9
	Internet and social media		5
**Region**			
	Paris region		16
	Rest of France		4

### Interview Process

A researcher who is a graduate in public health (PhD candidate) and trained in interview techniques conducted the semistructured interviews with professional experts. Each interview began with a presentation of our research subject and key associated concepts. The interview guide was not constructed based on a specific theoretical framework. Open-ended questions organized in a convergent manner were used to possibly prompt the interviewee on a subject. The content of the questions was based on factual information–seeking only. The semistructured interviews then followed the guide (see Multimedia Appendix 3) with adaptation depending on the interviewee’s experience. The interview guide was structured in four main sections: (1) features of experts, (2) youth sexuality concerns, (3) mechanisms for seeking information or exchange of experiences, and (4) opinions and experiences on web-based interventions and participatory features as peer education.

Interviews were audio-recorded after having obtained the agreement of the interviewed expert. The interviews lasted between 45 and 141 minutes with an average of 63 minutes. Most of the interviews were with individuals; only one interview was held with two experts working together. Interviews were carried out by telephone or at the experts’ place of practice, allowing the expert to remain in his or her working environment to complete the interview.

### Regulatory and Ethical Aspects

The study obtained a favorable opinion (no. 18-515) from the Institut National de la Santé Et de la Recherche Médicale (INSERM) Ethics Evaluation Committee (IRB0000003888) and was reported to the INSERM Data Protection Officer. Identifying information was anonymized in the transcripts. Expressed consent was given orally at the beginning of the interview, as was the authorization to record.

### Analysis

The interviewer transcribed and coded the digitized interviews verbatim and the notes taken during the interviews using N'Vivo 10 software. Using an inductive theme identification process to generate codes, an analytical framework was created. To ensure the validity of the results, the thematic analysis was carried out by two authors (PM, ELR), who followed the recommended phases and steps for the development of the themes in terms of qualitative content and thematic analysis [24]: Initialization, Construction, Rectification, and Finalization.

## Results

### Main Themes and Subthemes

Experts’ opinions were structured according to five main themes: (1) internet intervention; (2) sexual health; (3) internet skills and uses, and need for moderation; (4) multifaceted peers; and (5) minority peers. These themes are complementary, connected, and should be considered together for the development of participatory internet-based and sexual health promotion peer interventions. The themes and subthemes are presented in Textbox 1 (also see Multimedia Appendix 4 for the most significant quotes used to develop these themes).

**Key themes and subthemes identified in the interviews.**

**Theme 1: Internet intervention**

- Complementarity with existing offline sexual tools
- Secure, valid, and credible content
- Online personalized, interactive, and participatory features
- Adapting to rapid obsolescence of preferred media
- Social marketing to understand uses and preferences

**Theme 2:** **Sexual health**

- Importance of sexual health for young people
- Evolving sexual health concerns and issues

**Theme 3: Internet skills and uses, and the need for moderation**

- Heterogeneous internet use
- Diversity of skills in online information–seeking
- Need for online anonymity for sexual issues
- Risks of surfing the internet and social media
- Moderation of online social interactions
- Nonreceptivity of institutional messages

**Theme 4:** **Multifaceted peers**

- Importance of peer group for young people
- Peer education concept
- Notion of peers
- Peers’ involvement
- Limits of young people as “peer educators”

**Theme 5: Minority peers**

- Online peer group dynamic
- Need to find peers outside the neighborhood
- Inclusiveness in health promotion interventions
- Risk of stigmatization and discrimination
- Self-rejection as a determinant of participation
- Collaboration with specialized organizations

### Internet Intervention

In view of young people’s daily internet use, the majority of experts (18/20, 90%) believe that web-based participatory interventions to promote youth sexual health are attractive, with essential prerequisites. One expert found the intervention to be attractive without suggesting any limit. Another did not find the intervention to be attractive (owing to too many limitations).

#### Complementarity With Existing Offline Sexual Tools

A program coordinator insisted that a web-based action must be complementary to existing offline sexual health tools and actions (ie, the internet does not replace human contact; expert S5). Another highlighted the need to “digitize” existing health promotion techniques (S17).

#### Secure, Valid, and Credible Content

An expert in direct contact with youth indicated that to address young people through a web-based intervention, she will need to be sure that it is well embedded and disseminates valid and credible contents (S13).

#### Online Personalized, Interactive, and Participatory Features

Three experts (S1, S15, and S17) insisted on the importance of using internet tools to develop participatory actions, to go beyond observing or receiving information. Two experts (S1 and S17) mentioned online participatory features as attractive components, including shared construction of knowledge, the possibility of including influencers (role models), serious games, and chatbots (artificial intelligence). Two other experts (S8 and S19) considered that young people may have needs for health services offline beyond the internet, particularly in sexual health (eg, access to abortion, protection from violence). They also suggested providing geolocalized resources to allow for a personalized response, according to participant location.

For an internet site, it is important to be able to say, for a local area, where I can get more information, where can I get condoms? It must be locally sited and rooted in an area.

S8

#### Adapting to Rapid Obsolescence of Preferred Media

One expert (S5) warned that young people’s preferred online media sources are evolving quickly. Therefore, it is necessary to adapt actions to the evolution of internet uses and the rapid obsolescence of these preferred media.

It will already be obsolete, and will no longer correspond to their favorite network. This is something that changes fast, so for it to be set up and be effective, we have to be reactive.

S5

Two experts (S5 and S15) also proposed to use multiple online media sources in parallel (interconnected), to observe preferences, and to be as close as possible to media uses.

#### Social Marketing to Understand Uses and Preferences

Moreover, a communications specialist pointed out the need for a social marketing strategy to understand young people’s internet usage and to adapt to their preferences in implementing actions.

How to attract young people? It has to be a brand, there must be a marketing strategy, one has to think about several different sites. What we are studying is peer education, which is quite well known. But one just has to get on terms with different internet sites and know how to make them work.

S15

### Sexual Health

#### Importance of Sexual Health for Young People

Based on her research, a sociologist expert on young people’s internet usage and sexuality (S19) explained that among other health issues, those related to sexual health appeared to be the most important among young people. Three experts (S1, S9, and S11) emphasized the emotional dimensions of sexual health: love and sentimentality, as well as sexual relationships are central for young people. In this sense, three experts (S1, S2, and S4) also recalled the importance of a holistic approach to sexual health action, treating it in a global way and going beyond the prevention of risks.

#### Evolving Sexual Health Concerns and Issues

A sociologist expert on young people’s sexuality as manifested online (S16) emphasized the importance of taking into account, when planning actions, the evolution of a young person’s sexuality concerns, especially following their sexual debut. He recommended recognizing that internet use evolves with life trajectories: “Internet usage is linked to one’s situation, and depends on age and on the changing concerns implicated in one’s emotional and gender relations” (S16).

### Internet Skills and Uses, and the Need for Moderation

The majority of the experts (19/20, 95%) discussed young people’s internet usage as a route to address sexual issues (preferred media, use of social media to interact and search information). They emphasized the heterogeneity of the young population.

#### Heterogeneous Internet Use

One sociologist (S16) explained that exploring the internet may be a solitary activity at first when seeking to understand sexuality; in this phase, young people do not necessarily want to interact with others. However, they may ask questions on online search engines to find information and observe the exchanges in forum discussions (S3, S16).

Early adolescence, before the first sexual relations, is often a time of very solitary exploration of the internet, it is not social networks that are the most important. But at this stage young people do follow forums [discussions online].

S16

#### Diversity of Skills in Online Information–Seeking

Based on her experience, another expert (S19) insisted that heterogeneous internet usage for information retrieval must be considered. There is a diversity of backgrounds and different skills in research, analysis, and critical thinking concerning online information:

Young people from better-off backgrounds, who have the greatest inclination and also the most social, educational, economic, and cultural capital, will be the ones who will make the most use of the different resources the internet has to offer.

S19

On the same subject, four experts in direct contact with young people (S2, S3, S9, and S11) made the point that they are eager to find answers online and are not always critical as to the reliability of the source. However, two experts in direct contact with young people (S6) and in sociology (S16) considered young people to be sufficiently competent to screen online information.

Young people have quite a strong tendency to resort to the internet, and their grasp of technology enables them to know the difference between a site which gives valid advice and one which looks untrustworthy.

S16

#### Need for Online Anonymity for Sexual Issues

Moreover, given the intimate and personal nature of the topic of sexual health, three experts (S5, S6, and S19) mentioned the advantage of online anonymity as protection for those needing to ask questions or seek information. In practical terms, one explained the advantage of this: “They have anonymity already, so they will be able to ask their questions more easily than face to face, from behind their screen…without embarrassment or fear of judgment by their peers.” (S5)

One expert (S19) complemented this notion by explaining that young people generally leave their usual media sources that can identify them to go to other sites so as to have anonymity for questions on sexuality. However, two experts (S6 and S9) also pointed out that anonymity could have drawbacks, with the risk of cyberbullying: “It can also be a protection for the most abusive or malicious, the internet ‘trolls’.” (S6)

#### Risks of Surfing the Internet and Social Media

Five experts in direct contact with young people (S2, S6, S9, S10, and S13) were those who gave the strongest warnings about online risks such as access to unreliable and invalid information (S3, S9), exposure of bodies by “nudes” (S2, S3, S10), or access to pornography (S2, S10).

In schools and colleges there are problems to be dealt with which arise on social media, on Snapchat. We have had quite a lot of trouble with photos where girls are posing on social network sites.

S3

#### Moderation of Online Social Interactions

To address these risks, the majority of experts (15/20, 75%) expressed the view that online moderation is necessary, notably in web-based actions allowing social interactions. Even in a peer education intervention, educators should not disengage from their adult role (S7, S8). This moderation must make it possible to reduce hurtful acts and limit false information (S2, S3):

The moderator must be really good, so that as soon as there is a false statement, or one that is hurtful or insulting, the moderator intervenes. There would need to be a super-present moderator.

S3

Nevertheless, two experts (S7 and S16) thought that young people might consider this moderation as imposed from the outside, thereby losing the desired “between young people” aspect.

#### Nonreceptivity of Institutional Messages

More generally, three experts (S6, S7, and S16) noted that young people are not attracted or receptive to traditional prevention actions and messages developed by institutions. Some experts (S5, S11, S15) pointed out that existing sexological tools (school interventions, websites) are effective. In contrast, two experts (S7 and S16) were critical of young people’s perception of formal actions, which are considered to be not effective or too institutional (S7).

To address the nonreceptivity to institutional messages, two experts (S6 and S17) insisted on the need to involve young people in project reflection. Moreover, a teacher (S3) explained the importance of “peers” for the acceptance and integration of the information received: “We know that studies show that when information and knowledge are offered by a member of a peer group, it is better accepted and retained than when it is provided by the teacher.”

### Multifaceted Peers

#### Importance of Peer Group for Young People

For a sociologist expert on young people’s sexuality online (S19), the peer group corresponds to one of the new spheres of socialization where peers will be chosen and will take up a lot of space in the lives of young people. Peers could then intervene in education.

#### Peer Education Concept

Among experts who had experience in peer education (9/20, 45%), the majority (S1, S6, S7, S8, S15, and S19) considered peer education as part of an approach that involves youth participation in action. For another expert (S4), peer education was described as a discussion time between young people. One expert insisted on the importance of not considering young people only as “action users,” but rather including them in all stages of action development, not only in design:

This is a group of young people who self-select to set up a project which can be designed and made available to young people who are like themselves. [….] They are the ones who will take the initiative, and will be involved in the design, the implementation, and the evaluation.

S6

#### Notion of Peers

Six experts (S4, S6, S7, S8, S15, and S19) questioned the idea of “youth” being peers and the notion of “peers.” They insisted that peers must recognize and consider themselves as peers (S6, S7, S8, and S19). One (S6) advocated letting the group of peers form themselves within the action, without institutional involvement. One (S19) indicated that it is complicated to simply consider the “age” characteristic as the gateway. For one expert, young people are all peers and she considered this as a limitation: “It just means young people talking to young people, so they are ‘pseudo-pairs’.” (S4)

For others (S6, S8), it seemed more pertinent to define the notion of “peers” in terms of similarity in experiences or concerns, beyond the criterion of age:

“Similar” doesn’t mean in terms of gender or skin color, but similar in terms of daily realities of life. They will have unity in terms of place, geographical space, and age.

S6

#### Peers’ Involvement

Two experts (S10 and S16) addressed the issue of young people’s involvement in web-based peer education. Some could be leaders and others more passive: “Some young people will immediately want to position themselves as leaders within the group, and others will prefer to come and look and say nothing.” (S10)

Three experts (S6, S7, and S8) identified different peer functions: moderators, educators, and receptors. One (S6) believed that peers could achieve online moderation: they are vigilant and autonomous for self-regulation. He suggested identifying “peer moderators” when the group is formed, based on peer involvement.

#### Limits of Young People as “Peer Educators”

In this sense, some young people could be selected to be “peer educators” (with young people taking over the action). However, three experts (S6, S7, and S8) highlighted the limitation of training peers to become “educators,” considering that they would only become institutional messengers (S7, S8) or reproduce the same effect as the prevention facilitators (S6).

If the peers have been formatted by the institutions, they will become outsiders to the group. The group will quickly realize that there is an institution behind them, and they will keep away…because as soon as adolescents are transformed into health educators, they are no longer adolescents, they are spokespersons for the adults. They are spokespersons for approved messages. That’s what I call parrots, the faithful repeaters of adult speech.

S7

Yet, for one expert (S8), peer educators can facilitate close relationships, and people will believe information from peer educators because they are trained. For this expert, such peer educators may also have an effect on their social environment beyond their peers.

Experts were then divided between the need to let young people take over the action, with the right to their imperfections (S7), and the need for institutions to moderate and validate information and exchanges.

### Minority Peers

#### Online Peer Group Dynamic

Based on his experience, a sociologist expert on young people’s sexuality online (S16) explained that online peers are mainly the same people as physical peers but are also those engaged with for online interactions. Online social life is not generally separated from daily physical life (S19), with one exception:

For adolescents, it is rare to have a group of friends online which is completely different or much larger than one’s physical group of friends, but there is one exception to this which I think is important, and that is the case of sexual minorities.

S16

#### Need to Find Peers Beyond the Neighborhood

Some specificities could lead people to search out peers in online areas (S16). Sexual minority populations have specific needs, including finding peers on the internet and far from their immediate geographical area (S10, S16, and S19). In particular, lesbian, gay, bisexual, transsexual (LGBT) peers would be present, but outside of the immediate environment. For these young people, “online peers” are then part of their real lives and are not to be considered as “virtual” (S19): “In LGBT contexts, this is something we often find: accessing the internet to get in touch with a network which can’t be located in certain geographical areas.” (S19)

#### Inclusiveness in Health Promotion Interventions

To adapt the action to specificities, three experts (S15, S18, and S19) mentioned inclusiveness issues to be considered to take vulnerable populations into account (eg, LGBT, people with disabilities, overweight, or deaf). For one expert (S15), representing minority populations in mainstream communication actions is a way to be inclusive. Another (S19) addressed the issue of “ourselves” and the need to form more specific subgroups: “We need to be inclusive in all our statements, at the same time it is good to form subgroups and to offer services which also correspond to within-group expectations.” (S19)

The difficulty of being inclusive was underlined, as it requires significant material, human, and financial resources (S18) to have an intervention that speaks to all (S15).

If we want to be inclusive, then young white heterosexual girls will have to come up against queer people, young men who have sex with men, and come to terms with different life experiences.

S15

#### Risk of Stigmatization and Discrimination

Moreover, one sexual health communications expert (S15) explained that risks of discrimination and stigmatization of specific audiences can occur in an all-audience activity. Minority people could perceive stigmatization and feel excluded, looked down upon, or treated differently.

For HIV, the recommendations are a bit different. For example, for a heterosexual person, testing is needed at some point during one’s lifetime, whereas for MSM it has to be 4 times per year. So there one has to work carefully, because there begins to be a risk of stigmatization.

S15

#### Self-Rejection as a Determinant of Participation

One expert (S15) raised the problem of self-rejection affecting participation in a health program. A person who does not accept themselves will not want to be part of a peer group and take part in an action addressing issues of sexuality.

In the case of young MSM, some will not be at all willing to come near a peer-led health education program in which there is a risk of even raising the idea that desire for other men exists. There may be a kind of self-rejection.

S15

#### Collaboration With Specialized Organizations

To address discrimination and self-rejection, one expert (S15) raised the importance of collaborating with specialized associations/organizations to take into account points of view and specificities. These organizations could intervene to moderate online peer exchanges (S19).

### Proposals Derived From the Themes

From the thematic analysis, several proposals could be drawn out, which are presented in Textbox 2, that may be of direct use in designing an online participatory intervention for peer sexual health promotion.

**Advice from experts for intervention conception and evaluation.**

**Domain 1: Peer intervention for sexual health promotion**

- Conceptualize in advance what is behind the terms “peer,” “peer-led,” and “peer education”
- Complement existing online and offline educational practices by listing existing sexological tools
- Avoid institutional formatting of trained peer educators, as peers should remain peers. Peer leaders can be identified within an already formed community and then recruited as peer educators
- Define a framework for the involvement (interaction and participation) of young people
- Design a plan for facilitating and moderating peer exchanges
- Take a holistic approach to sexual and reproductive health, going beyond risk
- Identify in advance the specific needs of the populations
- Develop an inclusive, nonheteronormative approach that avoids stigmatization/discrimination of specific minority populations
- Involve local actors and associations to be as close as possible to the expectations of young people (with ability to provide answers in the moderation of exchanges)

**Domain 2: Internet support**

- Develop a secure online environment that fosters “self-confidence”
- Use online media that allow horizontal transmission of information (peer education), rather than top-down information systems
- Develop a brand image that is not institutional and that allows young people to recognize it in the online environment (use of social marketing techniques)
- Offer participatory (games, quizzes) and interactive (discussion forum, chatbot, questions and answers, possibility to contact a professional) features
- Integrate young people’s favorite influencers and online characters, and the possibility of interacting with them
- Propose individual spaces (messaging, information folders) within the intervention to take into account the needs of solitary exploration
- Ensure anonymity of participants to encourage youth participation in intimate issues
- Grasp “youth culture” to be as close as possible to digital and online uses
- Be responsive in understanding the preferred tools and their use in an intervention
- Propose an interconnection of online media (website and social networks) to retain young people, integrate them into daily use, and observe preferences
- Provide a geolocalized response for access to sexual and reproductive health services or to meet with resource professionals offline and close to people’s homes

**Domain 3: Conception and evaluation**

- Use the community-based participatory research model to involve all stakeholders at all stages of the project (bridge between research, field, and realities)
- Ensure the diversity of the peer group involved in the design and facilitation of the program to move beyond heterogeneity and integrate/represent minority populations (LGBTQ, deaf, disabled, overweight)
- Design a theoretical model to evaluate the effect of the intervention on:
  - Determinants of behavior change: knowledge, attitudes, literacy level, behaviors
  - Measure the effect of collective determinants on each of the determinants of behavior change: effects of online social interactions, perceived online social support, and online social capital
  - Analyze online peer social networks
- Define an operational framework for the online intervention, to define in advance the process indicators to be evaluated:
  - Journey within the youth intervention: those who are active or lurkers within the intervention
  - Most used features and tools
  - Number of visits, interaction and participation rates in the proposed activities (to be linked to the effectiveness evaluation)

## Discussion

### Principal Findings

This study is the first to highlight experts’ opinions on key points and requisites for developing attractive and acceptable peer interventions on the internet for youth sexual health promotion. Experts considered this kind of intervention to be attractive, but warned of inherent risks and limitations. The experts interviewed provided very concrete and useful advice for developing web-based interventions for peer education, specifically in the field of sexual health.

One of the strengths of this work is that the experts did not confine themselves in the very purpose of arguing their “professional” point of view. By contrast, they envisioned the intervention by considering all the other actors involved, especially young people, with the help of their field experience. The themes addressed are therefore very broad, allowing the intervention to be thought of in its different dimensions with a global vision. This positioning of the experts is a very good indication of feasibility for the intervention because it shows that the experts project themselves in a global approach.

These results raise the following points for discussion: (i) there is a need to understand online uses and risks to take advantage of the internet; (ii) if peers are integrated into participatory education, they must recognize themselves as peers and must be selected for other characteristics than merely age; (iii) the notion of peers and specific audiences still needs to be understood to be inclusive in the web-based intervention.

### Understanding Online Uses and Risks to Take Advantage of the Internet

Young people are daily users of the internet, but the experts stressed their heterogeneous skills in seeking information about sexuality, depending on sexual development. To adapt a web-based intervention for sexual health promotion, they highlighted participatory features but also underlined the need to provide geolocalized resources. They also emphasized the need to moderate content and exchanges. Understanding users by using social marketing and managing risks should make it possible to offer attractive, secure, and adapted interventions.

Grasping and understanding “youth culture” would make it possible to adapt health promotion actions to be as close as possible to users. Social marketing enables effective educational programs to be developed based on scientific knowledge and good communication [25]. To be reactive when faced with evolving preferences, it is advisable to design an interconnection of different media sources (eg, websites, apps, social network sites) and to offer attractive components (eg, games, individual spaces, forum discussions, or contact with influencers [26]). Important factors include the structure of the campaign and the content of the message (imaginative, fun, accessible, noninstitutionalized brand image, and engaging) [27].

However, young people may have difficulties in finding locally relevant information on health services [28]: needs may be expressed offline, and young users face many barriers of confidentiality, cost, and access to health services [29]. One solution is to adapt to target population environments [30] by providing geolocated information about access to offline services close to home. One example of this approach is the Australian organization PASH [31], which offers available local resources, including on-call resources, for personalized information.

To cope with online risks (eg, cyberbullying, pornography, body exposure), moderation should control interactions and abusive content, and provide a secure space that maintains the quality of information [32], while leaving space for user-generated content. For example, individuals may report a greater intention to participate in an online community that shows signs of moderation [33], but not if this moderation is involved too early [32]. A moderator should have an engaged and an interactive presence, and should be designed to generate new interests, provide discussion material, and respond to user requests [32]. The paradox is then to enable this moderation while at the same time allowing young people to be fully involved in the action.

### Integrate Peers in Participatory Sexual Health Promotion

Facing the lack of focus on institutional messages, experts recognized the value of peer integration in web-based interventions. However, when discussing this possibility, many experts warned of limitations: peers should not be mere messengers and should remain peers so as not to be seen as outsiders by the group. The challenge then lies in young people taking ownership of their own education. One solution evoked was to identify active peers who can take the role of peer leaders once the online group is formed. Some experts thought that young people should be included at all stages of the project.

For sexuality education using digital media, it is recommended to use a variety of interactive methods and nonformal settings [1]. The appeal of peer sexuality education is that it has always existed on an informal basis, with young people sharing information with each other, including personal experiences [34]. From an action perspective, young people must be allowed to identify themselves as peers, particularly through the formation of subgroups within the intervention. “Peer” education can appear at this time, since the feeling of being “among peers” influences participation.

From a peer-education perspective, we first need to define the degree of involvement of peers in different steps of the project so that it remains a “peer-led” intervention. Next, it is necessary to allow them, through informational or experiential exchanges, to develop their knowledge and skills (peer education) [8,9]. This is also about justifying the inclusion of peers [35]. Many online interventions mobilize peer interactions to promote sexual health [18,36-41]; thus, a framework should make it possible to define the types of interactions between peers and to clarify the knowledge or skills to be developed. Based on the attractiveness of social interactions, the challenge is then to move from this informal mode to formal and conceptualized action led by institutions, particularly on the internet. It is also important to conceptualize “peers” and to define the common characteristics required for peers to be considered.

### Understanding the Notion of Peers and Specific Audiences to Achieve Inclusivity

Experts do not always consider peers in the same way, with some prioritizing the “similar” aspect of a common level of life experiences, and others age.

Concretely, it would be interesting to consider the peer group as a nonfrozen and shifting network, built on common characteristics or paths, sharing not only an age characteristic but also sexual orientation, gender, health problem, or life experience. When considering “peer” education, we must define what this means, and when to differentiate “young people” and “peers.” “Adolescent” peer groups can be characterized by a high degree of social solidarity and a code that contrasts with adult values [7]. Nevertheless, peers can find each other more easily by way of experience. For example, the transmission of older people’s experiences of sexuality can sometimes establish a stronger link between peers of similar sexual orientation.

Considering this notion of peers, some young LGBT people, or those with disabilities or other characteristics, may feel excluded or unaffected by a general public action. For example, heteronormativity creates a sense of invisibility, invalidation, and marginalization for people of gender and sexual diversity [42]. Involving local actors or associations in actions permits an inclusive approach, to be close to specificities. Recommendations for inclusive research include using culturally appropriate language, not assuming that participants are heterosexual or that certain behaviors are “normal,” and being aware of one’s prejudices and knowledge limitations as a researcher [43]. In addition, community-based participatory research should integrate “all” target audiences into action management to elicit an appropriate response [22].

### Strengths and Limitations

The strength of our study lies in the fact that it is the first to analyze expert opinions on the potential web-based interventions for peer-to-peer promotion of youth sexual health. These opinions thus complement the results of the few existing studies showing the feasibility of this type of intervention. Beyond the practical aspects and first demonstrations of effectiveness, previous studies such as the Harnessing Online Peer Education (HOPE) study and peer-led, social media–delivered interventions have highlighted the major aspect of involvement of peer leaders [20,21], which we temper with our results (ie, peers must remain peers).

Moreover, our study proposes guidelines to design and implement this kind of intervention. The diversity of the experts interviewed makes it possible to obtain opinions from institutional, research, and field professionals. The analysis has made it possible to take into account expectations, realities, and obstacles on the sexual health, youth, education, and internet aspects.

One of our limitations is that we were unable to find researchers who had evaluated this type of intervention, since it has not yet been developed and evaluated. This would certainly have provided new methodological tools essential for action research. Despite this, we were able to interview developers of online sexual health content for young people who also worked on peer education. However, we did not interview peer educators who have participated in peer education programs and could have provided more information about this specific topic. This is an inherent limitation of our recruitment methodology. We could also have asked young people about their needs, expectations, and attractions for this type of intervention. This should be included when developing programs of action through participatory research.

### Conclusion

Experts expressed the view that web-based participatory interventions for youth sexual health promotion by peers must be tailored to sexual health needs (information-seeking, socialization, services), the evolution of internet uses, and preferences in terms of participatory features. This type of action requires youth involvement in an inclusive and holistic sexual health approach. Peer education can be implemented on the internet, but the quality of the intervention also relies on not making the intervention too institutional. Involving young people in their own education in an interactive, safe online space then has the potential to develop their empowerment and long-term positive behaviors, especially in the area of sexual health.

## Appendix

Multimedia Appendix 1 Consolidated criteria for reporting qualitative studies (COREQ): 32-item checklist.

Multimedia Appendix 2 Detailed table of experts’ characteristics.

Multimedia Appendix 3 Interview guide.

Multimedia Appendix 4 Detailed themes and key quotes from the qualitative analysis.

## Abbreviations

INSERM: Institut National de la Santé Et de la Recherche Médicale
LGBT: lesbian, gay, bisexual, transsexual
MSM: men who have sex with men
