# Supplementary material for: Expert Opinions on Web-Based Peer Education Interventions for Youth Sexual Health Promotion: Qualitative Study
Source: J Med Internet Res. 2020 Nov 24;22(11):e18650. doi: 10.2196/18650 (PMC7723739; doi:10.2196/18650)
Supplement: Multimedia Appendix 1 [file jmir_v22i11e18650_app1.docx]

Multimedia Appendix 1: Consolidated criteria for reporting qualitative studies (COREQ): 32-item checklist

| **Domain 1 : Research team and reflexivity** | **Personal characteristics** | |
| --- | --- | --- |
|  | 1.       Which author(s) conducted the interview? | Interviews conducted by PM |
|  | 2.       What were the researcher’s credentials? | MT: PhD candidate in public health; CA: MD-PhD; SG: MD; AB: MD-PhD; ER: PhD |
|  | 3.       What was their occupation at the time of the study? | PM: PhD candidate in public health; CA: professor of epidemiology; SG: researcher in public health; AB: university lecturer, researcher in public health ; ER: Research Director, researcher in public health |
|  | 4.       Was the researcher male or female? | 3 females, 2 male |
|  | 5.       What experience or training did the researcher have | Experience in conducting qualitative research (PM, SG), experience in interventional research surveys (PM, CA, AB), expertise in public health (all authors), expertise in sexual health (PM, AB, ER) |
|  | **Relationship with participants** | |
|  | 6.       Was a relationship established prior to study commencement | The interviewer did not know most of the participants before the study. Three professionals were colleagues by training. One participant taught a course in sexual health that the interviewer attended. |
|  | 7.       What did the participants know about the researcher? | At the start of the study, the aim of the research project, as well as the objectives of the study was presented. |
|  | 8.       What characteristics were reported about the interviewer/facilitator? | Interviewer characteristics were not reported to participants |
| **Domain 2: Study design** | **Theoretical framework** | |
|  | 9.       What methodological orientation was stated to underpin the study? | We used thematic analysis in a sociological theoretical approach. |
|  | **Participant selection** | |
|  | 10.   How were the participants selected? | Participants were recruited for their experience in sexual health, prevention and health promotion, peer education, youth health; in in direct contact with youth, at the level of research and institutions. We tried to represent a diversity of profiles and backgrounds. |
|  | 11.   How were the participants approached? | Originally by email |
|  | 12.   How many participants were in the study? | 20 |
|  | 13.   How many participants refused to participate or dropped out? Why? | 1 (reservation notice following participation in a high public health council) |
|  | **Setting** | |
|  | 14.   Where was the data collected? | In the participant’s office (11 interviews) or on the phone (9 interviews) |
|  | 15.   Was anyone else present besides the participants and researcher? | No. One interview involved two participants. |
|  | 16.   What are the important characteristics of the sample? | Diversity of backgrounds and occupation (see characteristics in table 1) |
|  | **Data collection** | |
|  | 17.   Were questions, prompts, guides provided by the author? Was it pilot tested? | The interview guide was tested, read and adapted during the interview according to the expertise of each participant. |
|  | 18.   Were repeat interviews carried out? Details | No repeat interviews. |
|  | 19.   Did the researcher use audio or visual recording to collect the data? | All interviews recorded. |
|  | 20.   Were field notes made during and/or after the interview or focus group? | Notes taken during all interviews. |
|  | 21.   What was the duration of interviews or focus groups? | From 43-141 minutes. Average: 61 minutes |
|  | 22.   Was data saturation discussed? | Data saturation was discussed with authors after 15 interviews. |
|  | 23.   Were transcripts returned to participants for comments and/or correction? | Transcripts not returned to participants |
| **Domain 3: Analysis and findings** | **Data analysis** | |
|  | 24.   How many data coders coded the data? | Two authors (PM, ER) created the initial coding tree using first samples interview. |
|  | 25.   Did authors provide a description of the coding tree? | The coding tree is the one presented in Table 2 and corresponds to the themes and sub-themes identified. |
|  | 26.   Were themes identified in advance or derived from the data? | The themes were derived both inductively and deductively |
|  | 27.   What software, if applicable, was used to manage the data? | Use of NVivo software. |
|  | 28.   Did participants provide feedback on the findings? | No feedback was obtained from participants |
|  | **Reporting** | |
|  | 29.   Were participant quotations presented to illustrate the themes/findings? Was each quotation identified? | We present some quotations to illustrate findings. |
|  | 30.   Was there consistency between the data presented and the findings | The data presented and the findings are consistent |
|  | 31.   Were major themes clearly presented in the findings? | We present the most important themes related to the study objectives in the findings |
|  | 32.   Is there a description of diverse cases or discussion of minor themes? | We report and describe diverse cases |
