# Supplementary material for: Expert Opinions on Web-Based Peer Education Interventions for Youth Sexual Health Promotion: Qualitative Study
Source: J Med Internet Res. 2020 Nov 24;22(11):e18650. doi: 10.2196/18650 (PMC7723739; doi:10.2196/18650)
Supplement: Multimedia Appendix 2 [file jmir_v22i11e18650_app2.docx]

Multimedia Appendix 2: Detailed table of experts’ characteristics

| **Acronym** | **Gender** | **Occupation** | **Organization type** | **Specializations** | | | | | **Region** | **Interview duration** |
| --- | --- | --- | --- | --- | --- | --- | --- | --- | --- | --- |
|  |  |  |  | **Peer education** | **Sexual health** | **Youth health** | **Education Prevention**  **Promotion** | **Internet** |  |  |
| **S1** | M | Professor of Public Health, Sexologist | Government agency | X | X | X | X |  | Paris | 87 |
| **S2** | F | Midwife, Nurse specialized in sexual health | Association |  | X | X |  |  | Paris | 46 |
| **S3** | F | College Teacher (Science and Sexuality Education) | National Education |  | X | X | X |  | Creteil | 43 |
| **S4** | F | Professor - Gynecology and Obstetrics | Public Hospital | X | X | X | X |  | Marseille | 53 |
| **S5** | F | Regional Health Education Program Coordinator | Public Hospital | X | X | X | X |  | Marseille |  |
| **S6** | M | Head of Prevention Department - Prevention project manager | Public Institute for Prevention and Health Promotion | X | X | X | X |  | Paris | 141 |
| **S7** | M | National (former) Head of the Social and Youth sectors, facilitator of the national professional network "young people on the move" | Association | X |  | X | X |  | Saint-Denis | 59 |
| **S8** | M | Professor of Health Promotion, Sociologist | Public scientific, cultural and professional Institution | X |  | X | X |  | Rennes | 47 |
| **S9** | F | Nurse specialized in sexual health | Public Hospital |  | X | X |  |  | Clichy | 43 |
| **S10** | M | Clinical Psychologist - Psychotherapist | Public Hospital |  |  | X |  |  | Provins | 54 |
| **S11** | F | Nurse specialized in sexual health (school nurse), Sexologist, Trainer in sexuality education | National Education |  | X | X | X |  | Creteil | 50 |
| **S12** | M | Prevention facilitator - Specialized educator | Public Institute for Prevention and Health Promotion |  | X | X | X |  | Paris | 47 |
| **S13** | F | Advisor in Social and Family Economy | Association |  | X | X |  |  | Paris | 54 |
| **S14** | F | Clinical Psychologist - Psychotherapist | Association |  |  | X |  |  | Anthony | 62 |
| **S15** | F | Social Marketing expertise Manager | Public Institute for Prevention and Health Promotion | X | X | X | X | X | Saint-Maurice | 70 |
| **S16** | M | Sociologist, University Lecturer | Public Research Institute |  | X | X |  | X | Pantin | 55 |
| **S17** | F | Documentation and Information Officer | Public Institute for Prevention and Health Promotion |  | X |  | X | X | Dijon | 54 |
| **S18** | F | Epidemiologist, In charge of studies and research | Public Institute for Prevention and Health Promotion |  | X | X | X | X | Saint-Maurice | 45 |
| **S19** | F | Sociologist, In charge of studies and research | Public Research Institute | X | X | X |  | X | Paris | 69 |
| **S20** | F | Social worker | Association | X | X |  |  |  | Paris | 72 |
|  |  |  |  |  |  |  |  |  |  | **Mean duration = 61 min** |
