# Supplementary material for: Expert Opinions on Web-Based Peer Education Interventions for Youth Sexual Health Promotion: Qualitative Study
Source: J Med Internet Res. 2020 Nov 24;22(11):e18650. doi: 10.2196/18650 (PMC7723739; doi:10.2196/18650)
Supplement: Multimedia Appendix 3 [file jmir_v22i11e18650_app3.docx]

**Multimedia Appendix 3: Interview guide**

*We would like to point out that the professionals interviewed did not have the same degree of expertise according to the different themes covered.*

*For some, it was not relevant to address certain issues, particularly methodological ones.*

*For others, we explored the questions further if the expert consulted was an expert on the subject.*

**PARTICIPATING FEATURES**

- What is your job?
- Place of exercise (establishment and geographical location)?
- In which population do you work (specificity, age group if possible)?
- What is the nature of your action for these young people?
- Have you ever been involved in implementing an action to promote the sexual health of young people? If so, can you describe this action and your role in it?

**THEME: YOUTH-SEXUALITY CONCERNS**

- What was your last exchange with a young person on the theme of sexuality?
- What are the recurring themes in your exchanges with young people (modulate according to the previous answer: is this a recurring theme, what are the other themes)?
- Insist if not answered: what do you think AYAs are interested in about their sexuality?
- Can you describe an action that you think best meets your needs? How did this one seem adapted to you?

**THEME: MECHANISMS FOR SEEKING INFORMATION / EXCHANGE OF EXPERIENCES**

- In your opinion, what are the sources that AYAs use to seek information about sexuality and to answer their questions?
- What do you think of the information found on the Internet?
- Do you think they take a critical look at this information?
- If social media not stated: What do you think of social media to disseminate information and share experiences about sexuality among AYAs?

**THEME: INTERVENTIONS, PARTICIPATORY ONLINE COMMUNITIES**

**Sub-theme: Existing interventions and experiences**

- For you, the Internet can be an intervention tool to promote the sexual health of AYAs?
- Are you aware of any sexual health promotion interventions deployed on the Internet for young people (if so, which ones)?
- If not said before: Have you ever participated in the development of an AYA sexual health promotion campaign on the Internet? If so, can you describe your involvement (if not already done before)? What were the opportunities for youth exchanges? Were there any exchanges with intervention professionals?

**Sub-theme: "Participatory online community" type intervention**

- Do you think that peer education (young people among themselves) on the Internet and social media would be a means of action to promote young people’s knowledge and good behaviors in sexual health?
- In your opinion, to what extent are interactions between young people on the Internet, discussion forums, blogs or social networks beneficial in promoting youth sexual health education?
- What would also be the risks and limitations?
- If the professional's participation in the design of an online participatory community (because said before in the experience question)
- How did you build your participatory youth community?
- If not answered in the previous question: What are the essential methodological points in the construction of this type of intervention (to be relaunched if more information on moderation, interaction, recruitment, animation, information and others is needed)?
- Have you encountered any difficulties in developing and implementing this action?
