# Supplementary material for: Expert Opinions on Web-Based Peer Education Interventions for Youth Sexual Health Promotion: Qualitative Study
Source: J Med Internet Res. 2020 Nov 24;22(11):e18650. doi: 10.2196/18650 (PMC7723739; doi:10.2196/18650)
Supplement: Multimedia Appendix 4 [file jmir_v22i11e18650_app4.docx]

**Multimedia appendix 4: Detailed themes and principal verbatims from qualitative analysis**

| **An Internet intervention** | **Complementarity with existing offline sexual tools** | « On the other hand, I think that it does not replace a face-to-face contact with a human and is a complement to the sexological tools. » (S5)  « I also think that we have school nurses who are very present and who are close to the students, we have social workers and guidance counsellors who are also very present.... The psychologists of the national education specialized in guidance [...] If the online groups are well moderated, etc... If the online groups are well moderated, etc. After being well advised by the National Education or even we could organize sessions here today we will use such and such a website such and such a tool such and such a network. » (S3)  « Yes, because everyone has different skills as well, and at some point they will have more need to go to sidainfoservice, then the hospital, to the forums... I think if it [sexual health education] is multifaceted, it also allows people to take what they need at some point in time » (S9)  « Nous pouvons voir, grâce à nos interventions, pour aller dans des collèges très différents, que les jeunes peuvent avoir des problèmes totalement différents (même au sein d'une même classe), et qu'on ne peut pas le voir derrière un écran. C'est pourquoi je dis que cela doit être complémentaire avec des actions sur le terrain, pour répondre vraiment aux besoins d'un jeune. » (S5)  « They [peers] are going to play a role, but in reality it will be more complex and more comprehensive strategies need to be put in place. For an internet site, it is important to be able to say, for a local area, where I can get more information, where can I get condoms? It must be locally sited and rooted in an area. » (S8) |
| --- | --- | --- |
|  | **A secure, valid and credible content** | « If I'm going to refer the young people I receive to an intervention on the Internet that deals with issues of sexuality, I'm going to have to be sure that the content is reliable and credible, that those behind it are reliable... the platform has to be safe as well. » (S13) |
|  | **Online personalized, interactive and participatory features** | « I think geo-localized resources are just essential for everyone. » (S19)  « For an internet site, it is important to be able to say, for a local area, where I can get more information, where can I get condoms? It must be locally sited and rooted in an area. » (S8)  « Today, there are no tools that have been properly developed, educational and inclusive in sexual health. Tomorrow there will be. So what tools do we have? We can have "game changers" or serious games that can educate through play about the different dimensions of sexual health, there can be artificial intelligence, and I'm thinking of chat rooms. We could have chatbot on the first times... first kisses, first sexual intercourse with someone [...] the first time could be a very good chatbot tool. A chatbot about pornography education, answering a thousand questions about pornography. A chatbot about desire. On desire there's a lot to say, on lack... so I think we can bring things to answer individual things through a panel of prepared questions. » (S1)  « These are people [influencers] who have such powers of communication that when they have their own interest and well they can direct, and correctly communicate, and this in a way that is much superior to the State, much superior to national education, because in addition, they are admired. But when you admire someone, you are submissive. And when one is submissive to someone one admires, one is in terror, one is afraid. So for me, a blogger that I'm going to follow, the risk is that I'll lose my critical spirit and at some point he'll tell me that he has beliefs that are not mine and I'll feel bad. So the internet... which is under the control of professionals whose job it is, whose job it is, well we're here for the people and not for ourselves. » (S1) |
|  | **Adapting to rapid obsolescence of preferred media** | « It's a very good idea, it's very interesting... Digital tools are interesting for the future, knowing that it's evolving rapidly. It will already be obsolete, and will no longer correspond to their favourite network. This is something that changes fast, so for it to be set up and be effective, we have to be reactive. I think that before you set up an application or a website, or Facebook or Instagram for example, it may already be obsolete and it will no longer be their concern and their favourite network. » (S5)  « A typical intervention (I don't know what form it could take) that is linked to several social networks (Facebook, Snapchat, Instagram), all the social networks used by young people. We would have to create something in common that would be visible on these different applications and these different networks. » (S5)  « It would be necessary to have an exhaustive vision of the evolution of Internet usage as it evolves. » (S16)  « She was telling me that she preferred Snapchat and Instagram... it was a good separation between her teenage world and the adult world, maybe that's why it's always evolving at the network level, because yesterday's teens have become today's adults, and I think it's a bit like the Facebook community. Maybe new teens need to use new media. I think Facebook is a little bit out of fashion among teens. I think back to a teenage girl who told me it was used by her mother, so maybe there's something to be heard there. » (S10) |
|  | **Social marketing to understand uses and preferences** | «It would be necessary to have a very exhaustive vision of the evolution of Internet uses as they evolve. If we had, for a sufficiently large group of young people facing prevention on the Internet, to see how their uses evolve in a very precise way. What are you going to see now that you didn't see before, but for that you would need to have a very precise list of the sites visited and the groups to which you belong. » (S16)  « You really have to look at the Internet as a tool and what we're looking at is peer education and that's something that's pretty well known... it's going to be the same thing on the Internet it's just a different place. What's complicated will be to master the environment because it requires a technological mastery. » (S15)  « You have to define before you know where to go what you want to do, share. You're going to need to be multi social network. If the goal is to interact in a group that is defined in advance I recommend Whatsapp, signal... Possibly a closed Facebook group. How do you get young people to come and yes you need to be a brand and make a marketing strategy. You have to think in several places. » (S15)  « We should be able to trace their route online. On social marketing issues we are not equipped. » (S15) |
| **Sexual health** | **Importance of sexual health for young people** | « Peer sexual health education is not disease education, sexual health is more than the physical act, it is the interaction of sexuality with other dimensions. » (S1)  « Sexual health is about learning about healthy wellness in its four dimensions [physical, social, mental and emotional], with the emotional at the center. When we said that, nothing can be done without the environment. » (S1)  « First of all, we're into the risks, benefits and pleasure of sexuality. We try to train them to deal with the positive aspects of sexuality, so as not to focus on the risks during the intervention. And over the years, this positive side of sexual well-being, as defined by the WHO, has been incorporated. It's something that was done gradually, and thanks to the planning, to professionals who brought me a lot of things that I didn't know. I had a vision of sexual health education that was quite old and risk-based, and we know that the programs that work are the holistic sexual health programs. » (S4) |
|  | **Evolving sexual health concerns and issues** | «There are those for whom it's part of everyday life, and there are others for whom it's a total blur... "No, but why are you talking to us about this, why are you talking to us about this, we're not interested yet. I tell them, "You speak for yourself, there are 13-14 year olds around you who started a long time ago. " » (S2)  «When I had studied very broadly the reasons why people go on the Internet in relation to their health and not just their sexual health, we could see that sexual health was quite high on the list of health concerns. » (S19)  «Internet use is very much linked to the position one finds oneself in according to age, and concerns evolve with one's position in love relationships, in gender relationships. Once these young people have had their first sexual relations, the questions change. The questions tend to revolve around contraception, other issues, risk, HIV, and so on. » (S16)  « We can see from our interventions, to go to very different colleges, that young people can have totally different problems (even within the same class). » (S5)  « In high schools there are much more experienced young people than in colleges. Some of them are shocked by you, and some are already having a hard time with it. So in fact there are some groups that are too heterogeneous in (younger) secondary schools. » (S2)  « There is the psychological and behavioural side which this year was much more developed than other years, with really precise key words: research, questioning one's sexual orientation, asking oneself the question of the image one sends to others, that the image is important for oneself as an adolescent, the obligation to be virile asked of boys. » (S3)  « What I could say, not to speak of one and only one young person, I would say that the issue of sexuality, it's quite in an evolution over the years. It can always be questioned even if it is not something that is described at the outset in a crudeness, because when faced with an adult, the teenager will tend to hide the elements of his sexual curiosity and sexual questioning. Afterwards, some teenagers will perhaps approach things more from the angle of a love encounter, even if the question of love is still to be questioned, we are talking about the feeling of falling in love or the question of sexual orientation, which may question some teenagers. » (S10) |
| **Internet skills and uses and need of moderation** | **Heterogeneous use of the Internet** | « Yes, many young people explain that at some point in their sexual trajectories, their socialization to sexuality, it [online social networks] becomes a place where they can ask questions. What we see is that despite everything, early adolescence, that is, before the first sexual relations, is often a time of exploration of the Internet, which is ultimately very solitary, and it's not through social networks that things are played out, but on the other hand, it's through forums. All the young people we've seen have already been to forums. However, no one ever posts on the forums. We use them very solitary, exploratory, without any dialogue. » (S16)  « I know they're going to go on the internet so I told them to be careful about the keywords they're going to enter on the internet because otherwise you're going to come across sites that are forbidden to under 18 year olds. » (S3)  « Early adolescence, that is to say before the first sexual intercourse, is often a very solitary time of exploration of the Internet and it is not through social networks that things are played out. On the other hand, we'll go to the forums. » (S16)  «What's really unique to adolescence, and to the young period, is the need to feel normal. And we're going to find that in the use of the forums. In other words, when my question has already been asked, it's because I'm not crazy, it's not just me, I'm not isolated and I don't have a specific problem. And all this will also allow the person to feel that, whatever his question, whatever his practices, whatever his situation, he will feel less isolated and less stigmatized by his question or by his living conditions, his behavior, his practices, his questions. » (S19)  « I know that they are going to look for information on the Internet, today it's a hyper-used medium [...] we prefer to tell them that there are sites that we know, that are reliable and recognized. » (S9)  « What is certain is that it is almost exclusively via the Internet, I don't see any other means at the moment... we're asking ourselves a question, it's on the Internet that we're going to see it. » (S10)  « It depends on the young people, some will talk very well about their sexuality, about the construction of their sexuality according to their questions, but others will very quickly evacuate this question because it is too intimate. On the contrary, they will consume very raw images but will be very modest about their sexuality. » (S10)  « We have a great diversity of situations but we often see that in general, the research that is carried out is carried out in slight anticipation, that is to say that it is rare to have young people who say that ... while they imagine their first sexual intercourse in high school, they rarely go into 6th grade to do very precise research on first sexual intercourse for example. But on the other hand, a few months before, we can see that there is a form of anticipation: it's likely to happen in the coming months, so I'm going to do this research at that time. » (S16) |
|  | **Diversity of skills in online-seeking information** | « On the one hand, there is the question of the dissemination of information, and it is certain that peer-to-peer education is easier, if young people are trained or made aware of knowledge in the first instance. This raises the question of who is the leader on the Internet. » (S8)  « For the internet, when you talk to them about search engines, well it's Google already, and then in the search bar they will type a question and therefore the importance of teaching them how to use internet tools, because they don't know how to use them well. » (S3)  « There is indeed a gap between individuals in the social dispositions to be able to sort information where young people from the most affluent backgrounds who have the most disposition and social, educational, economic, and cultural capital, will be the ones who will make greater use of the different resources that the internet can offer, crossing information by going to specific sites, while young people with fewer dispositions will go to the first three Google hits to do their research. » (S19)  « We are obliged to tell them that there is not only Google, to put keywords, to pay attention to the source, and it is true that they are very impatient, there is a very fast aspect of research on the Internet. "I see the first link, I click on it" and finally they don't look at the name of the website which can already point to the reliability of the source. » (S3)  « It is not only a question of reliability, but also of the diversity of information. On the one hand there is reliability and that's one thing, they're all looking for reliability, and they're all questioning what's being said on the Internet, maybe a lot more than the adults are. I really think it's looking for information and finding information. » (S19)  « There is a selective sorting of information found on the Internet among young people, particularly on online discussion forums. » (S8)  « The hard question of the ability to search, to discriminate information, to categorize, to classify information, to be wary of bad information, pseudo medical ... It's the same thing, teenagers and young adults are not necessarily trained to do this. When I see the way young students do research on the Internet and rush headlong into any data, any information because it's on the Internet. It sucks if they go looking for health information. » (S7)  «They are sometimes told to beware of the forums, as there may not necessarily be good information [...] it can be a bit worrying for them these false informations.» (S9)  « There is a tendency to underestimate the critical capacity of young people on the Internet, to think that they take all information for granted, that only those who have sufficient educational capital would be able to distinguish between things...what we see...is that young people have a fairly significant distance on the Internet, a mastery of technology that still allows them to know what a valid site is, a site that looks dubious.... » (S16) |
|  | **Need for online anonymity for sexual issues** | « Anonymity can be protection, certainly with other conditions, because anonymity can also be protection for unpacking things, but it can also be protection for the worst nastiness or the worst judgments, the "trolls" of the Internet. » (S6)  « You have to be able to be anonymous, it's better not to be recognized, especially in relation to teenage modesty... » (S10).  « They will be able to ask their questions more easily than in person, behind their screen. They can ask their questions without being ashamed or afraid of the judgement of their peers. » (S5)  « Anything we're a little ashamed to talk about because it calls into question either one's place in the peer group, or one's respectability, or one's practices, so sometimes it's easier to use the Internet to get the first answers. » (S19) |
|  | **Risks of surfing the Internet and social media** | « Since young people have smartphones, since they have Snapchat, they don't realize the impact, apart from the porn or sites and all that, but also the social life on the networks. » (S2)  « Access to pornography is commonplace, girls are more reserved about it, parents need to be able to verbalize with teenagers. All we know is that the earlier it is, the worse it is in terms of the construction of sexuality, it can become very damaging. And it's a lie to think that an image has no impact. Everything is image nowadays in the construction of young people. » (S10)  « Sometimes in college we have stories, problems to be dealt with that are born on social networks, on Snapchat, "oh but that's her, she put her picture like that. " " Yes, but you posted it " etc. after the story gets bigger and bigger. In college we had a lot of stories about girls posing in photos on social networks and we had it handled when it all started on social networks. » (S3)  « We had cases in 7th grade of students filming each other, but it wasn't on social networks. The last story was maybe five years ago. He would go to one of the students' homes and film himself naked. I don't know what they were planning to do with the video if we in the school know about it. But in any case, we in sex education feel that something had to be done. From the 6th grade on, we work on the image, cyber-harassment, cyber sexism, we also put a diaper back on in the 5th grade, we can say it's really young, but on top of that, this thing happened twice in the 5th grade with different kids, yes, there are definitely things going on between students. There are those who are very pro on the issue but there are also those who put themselves in danger. There is a whole panel of young people. » (S3)  « I saw a story about a 10-year-old girl, already trained, who sent a picture of her bare breasts to her boyfriend who posted it on Snapchat. Prevention has to start much earlier... but how are you going to do prevention in a heterogeneous group, in fifth grade, before they go to college, before it starts, without shocking more than half of them? » (S2)  « Anonymity can be protection, certainly with other conditions, because anonymity can also be protection for unpacking things, but it can also be protection for the worst nastiness or the worst judgments, the "trolls" of the Internet. » (S6)  « It is also new ways of getting in touch, even sometimes to enter into an almost sentimental relationship, where the body is more exposed, especially in fragile young people. » (S10)  « I am thinking in particular of social networks or rather the exchange of videos and photos, knowing that some young people, because of their greater fragility, do not always realize what they are doing and the repercussions it can have on them. I really work with fragile young people, and when it comes to videos, photos, it's already gone very far sometimes, it's already young people met by the police, it's stories of child pornography, or exchanges in high school that rub shoulders with harassment afterwards but not necessarily. Some young people themselves deliver photos on social networks in closed groups but distributed to the group as long as they sometimes show themselves naked, it's also new ways to get in touch even sometimes to enter into an almost sentimental relationship, where the body is more exposed, especially among fragile young people. » (S10)  « There are many groups of young people around adolescent issues, for example scarification, suicide attempts, suicidal thoughts... some young people have already created closed groups. These are groups where some young people can post their thoughts, their state, and in what I could perceive (it is young people who revealed it to me), others help each other ... but what is complicated is that it maintains the difficulty that the young person encounters and it can even lead to encounters that are a bit limited. » (S10) |
|  | **Moderation of online social interactions** | « The moderator of the network should be really good, that as soon as there is a false, hurtful, insulting word, the moderator should intervene. There should be a moderator who is super present. » (S3)  « Finally, when you look at the different online discussions, the messages are pretty benevolent, it's pretty inclusive, usually at a point where someone has been insulted or discriminated against or discredited, there's a point where the person comes back and says "thank you for supporting me". » (S6)  « On a rarement besoin d’intervenir car il y avait beaucoup d'autorégulation. L’autorégulation se fait beaucoup et assez facilement car je pense que les gens savent dans quel endroit ils sont. » (S15)  « If a girl asks how to get contraception, we'll answer right away by telling her to take the pill or something, but what the girl will expect is to be judged and to see how much she will be judged. So we anonymize her question, we pause it and see how people react. Often it's not bad because they are self-regulating, there is always a troll who says something stupid, there are people who say "yes, you're a bit stupid but no, there's no problem", we say what we said, and there is no medical emergency. There's always someone else who arrives saying "no, but that can make you sterile" and someone else who comes back saying "no, that's not true". » (S15)  « I think there's really a space for that, with young people who can talk to other young people... self-directed by offering a program […] » (S6)  « Moderation can be done a priori on the way in which discussion rules are set but always with the same pitfalls, i.e. these rules are perceived as imposed from outside. » (S16)  « Moderation can be done by peers, after what is complicated is that not everyone has the resources to always respond adequately. » (S19)  « The principle of the walkers of the net is to be integrated in the list of friends of a group where they can intervene smoothly to put there a little bit of information a little bit of common sense sometimes. » (S7)  « The main thing we have to work on is that the institutions should not be afraid of what it can lead to. Today the major reticence is that young people are saying things that aren't true. I think we're not looking at it the right way. The goal is not for them to provide good information, the goal is for them to motivate people like them to take an interest in an issue. » (S6)  « I think it might be a good idea... I don't know about the quality of the information, that there isn't any false information circulating or things that are a bit stigmatizing, it's rather that limitation that would scare me a bit... the whole exchange side, we understand each other among young people, I think it's really good, to talk about each one's feelings, their experiences, their experiences, how to do it, I think it's really good. What could frighten me is the limits of moderation and stigmatization. The Internet can sometimes be hard. » (S9)  « As long as it's supervised by an adult I would say yes, because the teenager still needs supervision, otherwise it would quickly fall into what we already see, the groups that already exist... so there is mutual help on the social networks. » (S10)  « Moderation can be done a priori on the way in which discussion rules are set, but always with the same pitfalls, i.e. these rules will either be perceived as being imposed from the outside, so we will no longer be too much into a peer-to-peer thing, or they will go through the peers, but through peers who will be perceived as agents of the institution. » (S16) |
|  | **Non-receptivity of institutional messages** | « Young people are not going to see what the institutions are proposing, I think that there is something to think about in terms of the fact that they are not able to capture these young people. » (S16)  « What I'm noticing is that even if there are prevention campaigns, they don't necessarily reach everyone, it's not possible, and some young people are not going to be in there, even if we give them a sexuality education course, they're going to completely miss out, because it's going to make them laugh or it's too intimate. » (S10)  « It is well known that, in order to do real prevention, we have to talk to young people before they are really concerned, and not wait until they are already in it, already experiencing it. » (S3)  « They will soon find out that there's an institution behind it and they'll run away. » (S7)  «Studies show that when information or knowledge is provided by a peer, by another student, it is better retained than when it is provided by the teacher. » (S3) |
| **Multi-faceted peers** | **Importance of peer group for young people** | « The peer group is going to start to take up space in their lives, they started to be on buddies they really chose. And the parents are going to take up less space and the space is going to be shared through the different spheres of socialization. » (S19) |
|  | **Peer education concept** | « A group of young people who designate themselves to set up a project that is conceivable and that they implement with young people who are similar to them, similar does not mean similar in gender or skin colour, but rather similar in terms of the daily realities of life. » (S6)  « If we have a shared construction of messages between peers, it is much more effective than the stamp of Public Health France, Unesco Chair or Directorate General of Health. What would be nice would be to have platforms with the partnership of the institution but led by young people. » (S1)  « Studies show that when information or knowledge is provided by a peer, by another student, it is better retained than when it is provided by the teacher. » (S3)  « They are the ones who are going to take the initiative, they are the ones who are going to design, they are the ones who are going to implement and they are the ones who are going to evaluate. » (S6)  « Two things: one the notion of invisible peer is interesting because it is perhaps there that we would have a in-between between the too even and the not even at all. Maybe that's where there is something there. Maybe there is something to be created on this invisible peer but taking care that it doesn't end up too quickly as the bearer of the good word, that's always the problem. Secondly, what we learn from those who are not like ourselves, I think that there is an adult responsibility in history, adults have a responsibility to contribute to adolescents and young people. » (S7)  « Yes, but there has to be a trigger, there has to be an interest, there has to be someone who has the right idea of triggering the thing, or by mutual agreement together, at some point they say to each other, we have to think about what we are going through and what is happening to us. The trigger has to be internal to the group. » (S7)  « They [peer educators] are the primary beneficiaries of peer education, since they are the ones who will be involved in the knowledge and training issues, and these are some interesting effects to see. Then we can say to ourselves, for the other peers, we reinforce inequalities, I would say yes and no because it allows access to a social existence that we would not know, there are other registers that will intervene. » (S8) |
|  | **Notion of peers** | « It's a peer education program, and I put it in quotation marks, because not all the users are the same age at all, it's just young people talking to young people, you might say, so it's pseudo-peers. » (S4)  « Similar does not mean similar in gender or skin colour, but rather similar in terms of the everyday realities of life. » (S6)  « It's more the question of the common characteristic, even if age obviously plays a role, but we are young people from local missions who are going to speak to other young people from local missions, high school students who are going to speak to other high school students. The idea of a framework of experience and expertise still plays an important role. » (S8)  « The acceptance of "peers" that we have today is that, it's a group of young people or something else that defines itself as a group, so for example, high school students from the same school, well, they're peers. They have a unity of place, of geographical space, of age... » (S6)  « The peer must recognize the one who speaks as a peer. And if the speaker is not from his or her culture or background or from his or her corner or language it does not work. » (S7)  « We could talk about gender identity, social belonging, shared life trajectories, et cetera. There are people who make a living out of it, ex-convicts. They're credible in the sense that they know what they're talking about. » (S7) |
|  | **Peers’ implications** | « It is the question of how a group is built, there are always leaders in a group, it always happens a little automatically. In a recruitment process, some young people will immediately want to position themselves as leaders within the group, and then others will no longer want to come and watch and say nothing as well. But for all that, get informed. You have to give each young person the freedom to take on the role he or she wants. » (S10) |
|  | **Limits of young people as “peer educators”** | « I have seen a number of peer education programmes produced by young people within the framework of a working group of the Ministry of Youth and Sport and each time the tools proposed were tools that were pure reproduction of the ineffectiveness of the adult campaigns. » (S7)  « If we train young people to do what we do, we don't complete, we do the same thing, done by young people, but globally, we do the same thing. If they are very well trained, they will do the same thing as our animators who do this on a recurring basis. If we really want to complement, we have to act on another lever. » (S6)  « I've seen it in health peers, every time it implies that the peer must be a model of perfection. Let's start by relieving them of that by telling them that they are entitled to their inconsistencies, their practices... Yes, perhaps in this case we can support the peer a little dynamic. But above all we must be careful not to restrain them and not to restrain themselves by preaching the good word. » (S7)  « If they are trained to think about health communication and they are trained to pass it on to their peers, they are no longer peers, that's the problem. You can make them think they are peers, but they are no longer peers. » (S7)  « If peers have been formatted by institutions, they quickly become strangers to the group. » (S16)  « What I have observed is that just because one is young, the other will believe us. It makes it easier to have close relationships, on the other hand, on health information, we will believe him because he [the peer educator] has been trained. » (S8) |
| **Minority peers** | **Online peer group dynamic** | « For adolescents, it is rare to have a group of friends online which is completely different or much larger than one’s physical group of friends, but there is one exception to this which I think is important, and that is the case of sexual minorities » (S16) |
|  | **Need to find peers outside the neighbour** | « Facebook is going to be the place where we're going to go on specific groups, we're going to, eventually meet people through that, and so yes, it's a place to expand the network. » (S16)  « In LGBT contexts, this is something we often find: accessing the internet to get in touch with a network which can’t be located in certain geographical areas. As a result, they are not virtual peers, that is to say that very quickly they will take up a lot of space, that because they are also other humans, that they are also incarnated, that it will also be possible and credible, that it will take up that place in the lives of young people. » (S19) |
|  | **Inclusiveness in health promotion interventions** | « I'm thinking of deaf audiences. There are many ways to communicate, there is sign language, there is writing, not everyone reads, you're going to have blind audiences..., to develop online accessibility, it requires means [...] everything depends on where you place the cursor on inclusiveness. » (S18)  « It would be super interesting to create an inclusive group where everyone could ask questions openly and where the others even if they don't feel concerned by the question asked, either they let it go, or on the contrary they say the answer because they are also interested even if it doesn't affect them personally. » (S3)  « But I think you can integrate... it's going to take a lot of work and attention from you, from the supervisors, in the co-construction of young people... in my opinion you can integrate sexual and sexist violence, some of the validist discriminations but not all of them [handicap], grossophobic discriminations must be able to be included and discriminations on sexual orientation and gender discriminations must be able to be integrated... with terms that must be accessible, we are going to have a public that is not going to have access to the exact terminology and the conception of discrimination and difference is also built on terminology... in fact, it would be almost necessary to bring this terminology back to them in order to be able to give them access to this diversity and inclusiveness in their words and conceptions... » (S18)  « I highly doubt it. I look at the long experience of AIDES. Adolescents who are most in difficulty in the future are in a close relationship with risk and most impervious to the good word, the professions of faith. This goes beyond the technique of communication. They see no future they have no self-esteem so they don't pay attention to them. » (S7)  « We need to be inclusive in all our statements, at the same time it is good to form sub-groups and to offer services which also correspond to within-group expectations » (S19)  « If we want to be inclusive, if there are only young heterosexual white girls who want to participate because they're interested in health and because they want to study nursing, and there are 10 of them, how are you going to bring inclusiveness into this approach? In other words, they're going to have to rub shoulders with queers, MSM boys for a while. They're going to have to take into account a different experience of the femininities of women in working-class neighborhoods, for example, they're going to have to confront different concepts that they're not necessarily used to. That's what we have to be attentive to. How to work with people who are really concerned. » (S15) |
|  | **Risk of stigmatization and discrimination** | « For example, for HIV testing, the recommendations are a little different. For example, for a straight person it is a lifetime test while for MSM it is 4 times a year. So there we have to play it a little fine because there is a risk of stigmatization, when we have populations that are more concerned by public health messages because they are more concerned by discrimination, by stigmatization, by marginalization due to precariousness. » (S15) |
|  | **Self-rejection as a determinant of participation** | «In the case of young MSM, it is not going to be at all attractive to approach a health peer education program where there is a risk of even evoking the idea that there is a desire for men. There is a form of self rejection. » (S15)  « They have to want to participate and they have to want to fight... » (S15) |
|  | **Collaboration with specialized organizations** | « Work with concerned associations that work on HIV, so we will work with African associations or MSM that are in fact associations of concerned people. In general, these associations "do" for but also "are" made up of concerned people, so that's what also allows us to have this approach... We think about our communications with these people, we can have messages that can be addressed to everyone. When we put condoms on, it's addressed to everyone, no matter who they are, and then we have to represent the people they feel concerned about as well. When we say MSM and when we say African migrants right away we think that it's not the only problem they have with HIV in today's society. So we will try to get closer to people through the associations we know and in places where they live so that this message more specifically to them alone is not seen too much by the rest of the world and that this message is seen mainly by the people concerned. » (S15)  « Adults from associations of specific populations can be found with whom to discuss how to integrate young people from these populations into an Internet-based peer education programme. » (S15)  « That was also the originality of our program, and that's why it worked, because we brought together the different professionals who were already doing sexual health interventions (in school we called it sexual education), family planning, general counselling, etc. and we created this free education with as teachers the people who were in the field, who were already doing that in the field, we used their experience to create peer education, by federating people around a project". Groundwork for curriculum development. » (S4)  « Adults from associations of specific populations can be found to discuss how to integrate young people from these populations into an online peer education programme. » (S15) |
